# Systematic screening of soluble expression of antibody fragments in the cytoplasm of *E. coli*

**DOI:** 10.1186/s12934-016-0419-5

**Published:** 2016-01-25

**Authors:** Anna Gaciarz, Johanna Veijola, Yuko Uchida, Mirva J. Saaranen, Chunguang Wang, Sohvi Hörkkö, Lloyd W. Ruddock

**Affiliations:** Faculty of Biochemistry and Molecular Medicine and Biocenter Oulu, University of Oulu, Oulu, Finland; Department of Medical Microbiology and Immunology and Medical Research Center, University of Oulu, Oulu, Finland; Nordlab Oulu, Oulu University Hospital, Oulu, Finland

**Keywords:** (3-10): Antibody fragments, Disulfide bonds, Fab, scFv, Cytoplasm, *Escherichia coli*

## Abstract

**Background:**

Disulfide bonds are the most common structural, post-translational modification found in proteins. Antibodies contain up to 25 disulfide bonds depending on type, with scFv fragments containing two disulfides and Fab fragments containing five or six disulfide bonds. The production of antibody fragments that contain native disulfide bonds can be challenging, especially on a large scale. The protein needs to be targeted to prokaryotic periplasm or the eukaryotic endoplasmic reticulum. These compartments are specialised for disulfide bond formation, but both compartments have limitations.

**Results:**

Here we show that the introduction into the cytoplasm of a catalyst of disulfide bond formation and a catalyst of disulfide bond isomerization allows the efficient formation of natively folded scFv and Fab antibody fragments in the cytoplasm of *Escherichia coli* with intact reducing pathways. Eleven scFv and eleven Fab fragments were screened and ten of each were obtained in yields of >5 mg/L from deep-well plates. Production of eight of the scFv and all ten of the Fab showed a strong dependence on the addition of the folding factors. Yields of purified scFv of up to 240 mg/L and yields of purified Fab fragments of up to 42 mg/L were obtained. Purified fragments showed circular dichroism spectra consistent with being natively folded and were biologically active.

**Conclusions:**

Our results show that the efficient production of soluble, biologically active scFv and Fab antibody fragments in the cytoplasm of *E. coli* is not only possible, but facile. The required components can be easily transferred between different *E. coli* strains.

**Electronic supplementary material:**

The online version of this article (doi:10.1186/s12934-016-0419-5) contains supplementary material, which is available to authorized users.

## Background

**Fig. 1:**
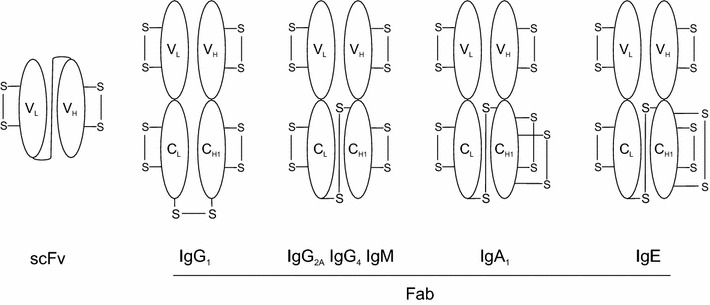
Schematic representation of antibodies fragments. Single chain (scFv) and Fab antibody fragments of types produced here are shown along with the position of the intra- and inter-molecular disulfide bonds

Antibody fragments, in particular scFv and Fab fragments (Fig. 1) have a wide range of uses in both academia and industry, including a critical role in diagnostics and an increasing role in therapeutics. While the recombinant production of full length antibodies is virtually exclusively performed in mammalian cell culture (for reviews see [1, 2]), in particular in Chinese hamster ovary (CHO) cells due to the requirement for post-translational modifications such as disulfide bond formation and N-glycosylation, a wider range of production platforms are used for scFv and Fab fragments which contain only disulfide bonds. These include production in the periplasm of prokaryotes, such as *Escherichia coli* (for example [3]), in the endoplasmic reticulum of eukaryotes including yeast, insect and mammalian cell culture (for examples [4, 5]) and in cell free expression systems (for example [6]). The lack of a clear frontrunner for a production system for antibody fragments reflects the fact that all have advantages and disadvantages.

Production in *E. coli* has a number of advantages over other systems, including low cost, rapid growth, high biomass, easily scalable cultivation and clear regulation for therapeutic protein production. The primary disadvantage of *E. coli* for antibody fragment production comes from the fact that production of the folded state can only occur in the periplasm as this is the only cellular compartment in *E. coli* in which catalysed formation of native disulfide bonds naturally occurs. The disadvantages of periplasmic production are twofold. Firstly the volume of the periplasm is much smaller than that of the cytoplasm, being typically cited as 8–16 % of the cell volume [7]. Secondly, the capacity secretion apparatus from the cytoplasm to the periplasm is easily overloaded, though this can be mitigated by reducing expression levels [8]. Both of these result in general lower levels of production of proteins in the periplasm compared with production in the cytoplasm.

To overcome these disadvantages we recently developed a system for the efficient production of disulfide bond containing proteins in the cytoplasm of *E. coli*, known as CyDisCo. CyDisCo is based on coexpression of a catalyst of disulfide bond formation, usually a sulfhydryl oxidase such as Erv1p [9, 10] but alternatively an inverted DsbB or VKOR [11], plus a catalyst of disulfide bond isomerization—either DsbC or PDI. CyDisCo has been used in house to produce more than 200 proteins, with the most optimal combination to date using Erv1p and PDI as the catalysts of native disulfide formation.

Here we addressed the question whether the CyDisCo system could be used to efficiently make scFv and Fab antibody fragments in the cytoplasm of *E. coli*. Specifically, we wanted to know whether this system was generic rather than specific i.e. if it would enable production of a wide range of antibody types. Accordingly we wished to have representatives of (i) different classes of antibodies (IgG_1_, IgG_2_, IgG_4_, IgA_1_, IgE and IgM were chosen); (ii) antibodies from different organisms (human, mouse and humanized were chosen); (iii) representatives of well-known and widely used antibody drugs (Humira, Herceptin and Tysabri were chosen); (iv) representatives of antibodies that arose from Finnish academia with potential use in diagnostics (Maa48, K2 and 3211 were chosen). Where possible we also wanted structural information on the antibodies to be available such that any differences observed in the efficiency of production could potentially be linked back to differences in structure. The results indicated that more than 90 % of the scFv and Fab fragments tested could be produced in the cytoplasm and that they were correctly folded and biologically active.

## Results and discussion

### Systematic screening of antibody and antibody fragment production in the cytoplasm of *E. coli*

Antibodies contain a large number of disulfide bonds in their native state e.g. an IgG_1_ contains 19 disulfide bonds. However, they generally have a regular pattern with one intra-molecular disulfide per domain plus inter-molecular disulfide bonds which link the heavy and light chains together and the two heavy chains together. Exceptions to this pattern exist, for example in IgA and IgE there is an additional disulfide bond in the first constant domain of the heavy chain (Fig. 1). The low number of disulfide bonds suggests that it should be possible to make natively folded scFv (two disulfides) and Fab antibody fragments (five disulfides, except IgA and IgE which have six disulfides) using CyDisCo in the cytoplasm of *E. coli*. These are especially attractive targets for production in the cytoplasm as they lack the consensus N-glycosylation sites found in the Fc region of antibodies.

We therefore decided to systematically screen the production of scFv and Fab antibody-fragments in the cytoplasm of *E.coli* using the CyDisCo system. Eleven antibodies were chosen for this screen, three human IgG_1_ (Humira; PDB structures 3WD5, 4NYL [12, 13]; Maa48 [14] and K2 [15]), two humanized IgG_1_ (Avastin; PDB structures 1BJ1, 1CZ8 [16, 17] and Herceptin; PDB structures 1FVC, 1N8Z, 4HKZ [18–20]), a mouse IgG_1_ (3211; An anti-BNP antibody, Veijola, Vuolteenaho and Takkinen, unpublished observations), a mouse IgG_2_ (PDB structure 1IGT [21]), a humanized IgG_4_ (Tysabri; PDB structure 4IRZ [22]), a human IgA_1_ (PDB structure: 3M8O [23]), a human IgE (PDB structure: 2R56 [24]) and a human IgM (PDB structure: 1QLR [25]). Each of the 11 scFv and Fab fragments derived from these antibodies were expressed from otherwise identical vectors and expressed and purified under identical conditions i.e. not optimized for individual proteins, such that antibody specific differences could be observed.

Previously it has been reported that some antibody fragments can be expressed in *ΔtrxB*/*Δgor* strains of *E. coli* (for examples [26, 27]) in which the reducing pathways have been removed, but which have no active pathways for disulfide bond formation [28]. This suggests that some antibody fragments may require little or no assistance from catalysts of disulfide bond formation to reach a soluble state. To examine this possible effect all 22 antibody constructs were expressed with and without CyDisCo components in 24 deep well plates using the KEIO collection parental K12 *E. coli* strain. This strain has the cytoplasmic disulfide bond reducing pathways intact. All expression tests were conducted at least in quadruplicate.

**Fig. 2:**
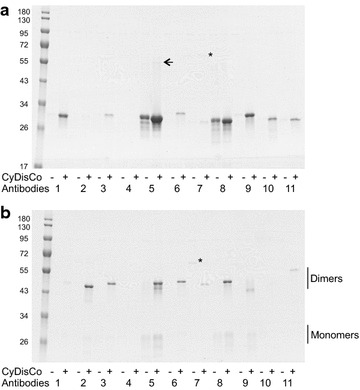
Purification of antibodies and antibody fragments expressed in DWP in the cytoplasm of *E. coli*. Representative Coomasie stained non-reducing SDS-PAGE analysis of NEM treated IMAC purified antibody fragments in the cytoplasm of a K12 *E. coli* strain with (+) and without (−) expression of CyDisCo components Erv1p and PDI. Expression in 24 deep well plates, EnPressoB media at 30 °C. **a** scFv. The position of a Herceptin scFv disulfide linked dimer is marked with an arrow; **b** Fab. The position of the Fab dimer and light chain and heavy chain monomers are marked. In both panels the order is molecular weight markers (1) Humira (IgG_1_), (2) Maa48 (IgG_1_), (3) K2 (IgG_1_), (4) Avastin (IgG_1_ humanized), (5) Herceptin (IgG_1_ humanized), (6) 3211 (IgG_1_ mouse), (7) 1IGT (IgG_2_ mouse), (8) Tysabri (IgG_4_ humanized), (9) 3M8O (IgA1), (10) 2R56 (IgE) and (11) 1QLR (IgM). All antibodies are human unless otherwise indicated. In both panels an E. coli protein which is occasionally seen co-eluting is marked with *. Treatment with NEM results in a slight smearing and laddering of the protein band. This is not seen in the absence of NEM (see Fig. 3a as an example)

Examination of the soluble lysates by SDS-PAGE followed by Coomassie staining allowed the direct visualization of antibody fragment production in high yields for many of the constructs with CyDisCo present (data not shown). Since densitometric analysis of yields from lysates is prone to error e.g. due to co-migrating *E.coli* proteins, the 22 constructs expressed in the presence and absence of CyDisCo components were purified by IMAC from 3 mls of culture grown in 24 deep well plates. Since disulfide bond isomerization can occur in SDS if a free thiol group is present, the purified proteins were treated with N-ethylmalemide (NEM) to block free thiols before being analysed by reducing and non-reducing SDS–PAGE. The results (Fig. 2; Table 1) indicate that 10 out of the 11 scFv and 10 out of the 11 Fab were expressed in sufficient yield to be visible in Coomassie stained gels (limit of detection circa 3 mg/L yield) when CyDisCo components were present. In contrast only two scFv, Herceptin and Tysabri, showed high-level CyDisCo independent production and no Fab fragments were purified in the absence of CyDisCo.

**Table 1:** Average yields of purified antibody fragments from DWP cultures

Antibody name	Type	scFv (mg/L)	Fab (mg/L)
Humira	IgG_1_ human	33	5
Maa48	IgG_1_ human	4	43
K2	IgG_1_ human	11	26
Avastin	IgG_1_ humanized	–	3
Herceptin	IgG_1_ humanized	271	50
Tysabri	IgG_4_ humanized	139	25
3211	IgG_1_ mouse	19	9
Mab123	IgG_2_ mouse	6	42
3M8O	IgA_1_ human	86	17
2R56	IgE human	32	–
1QLR	IgM human	26	9

Yields were determined by densitometric analysis using yields of purified scFv and Fab from shake flasks as calibration controls

All of the scFv produced were monomeric, with the exception of a very faint band of disulfide linked dimer for the Herceptin scFv present in some replicates. In contrast the Fab were predominantly a disulfide linked dimer of the heavy and light chains, though some monomeric light and heavy chains i.e. not disulfide linked, could be observed e.g. for Herceptin and Tysabri. When small amounts of monomeric purified heavy and light chains were observed in the non-reducing gel analysis the apparent ratio was always 1:1 suggesting that dimers of the heavy and light chain were purified, but that some of these lacked the inter-chain disulfide bond and so ran as monomers on non-reducing SDS–PAGE.

### Shake flask scale production, purification and analysis of antibody fragments

**Fig. 3:**
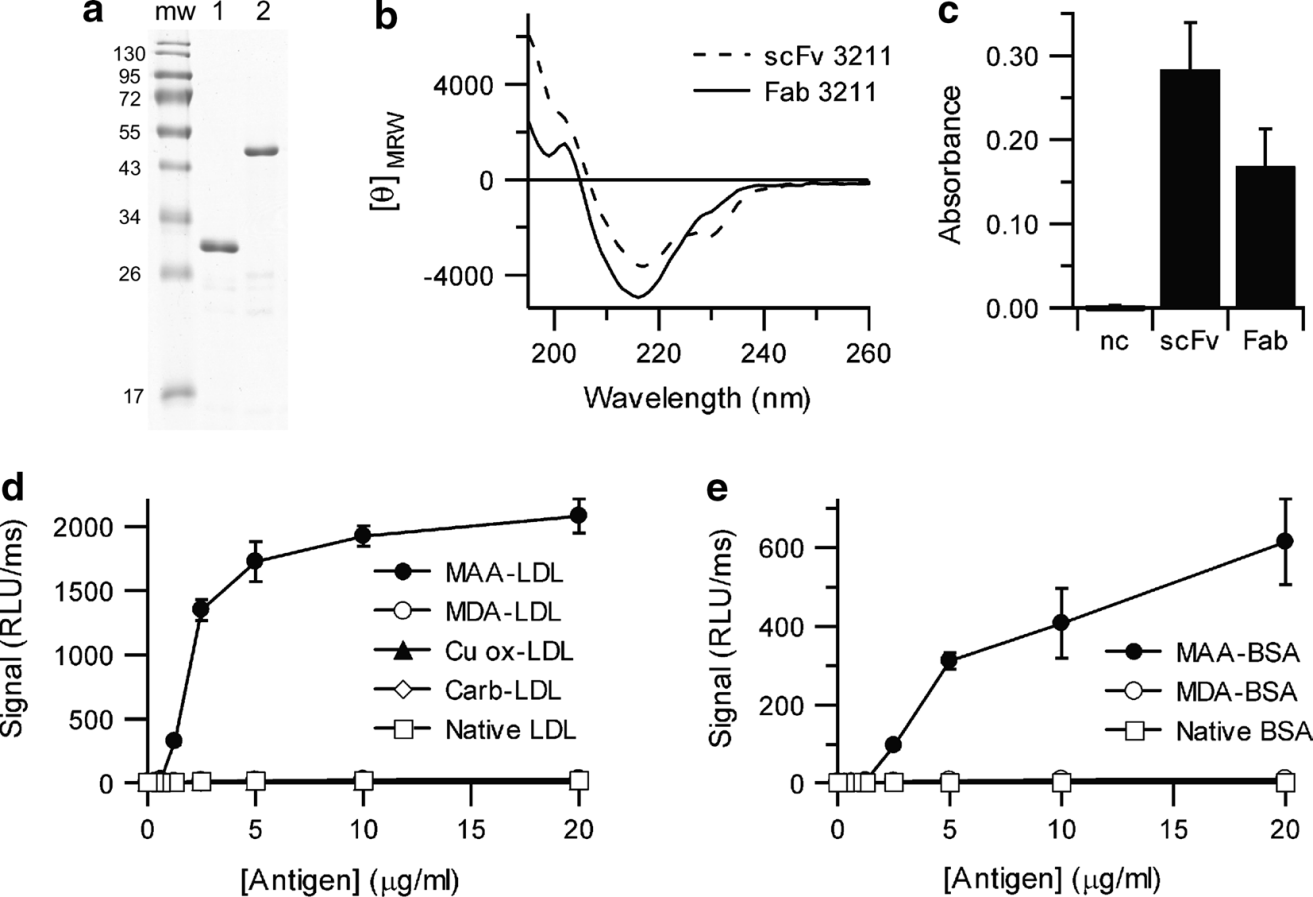
Analysis of antibody fragments produced using CyDisCo in the cytoplasm of *E. coli* grown in shake flasks. **a** Coomassie stained non-reducing SDS-PAGE analysis of IMAC purified 3211 (IgG_1_ mouse) scFv (*lane* 1) and Fab (*lane* 2) antibody fragments. **b** Far UV circular dichroism spectra of IMAC purified 3211 scFv and Fab fragments. **c** 3211 scFv and Fab binding to recombinant NTproBNP1-76; **d** Maa48 Fab binding to native and modified LDL; **e** Maa48 Fab binding to native and modified BSA

Small scale production in 24 deep well plates (DWP) allowed preliminary screening of the production of antibody and antibody fragments, but it did not produce sufficient protein for more detailed analysis. To examine in more detail the proteins produced using CyDisCo five scFv (Herceptin and Tysabri which showed CyDisCo independence, and 3211, 3M8O and 2R56 which showed CyDisCo dependence for production) and four Fab (Maa48, 3211, 3M8O and 1QLR) were chosen for production in shake flasks. Based on the results from DWP expression, Herceptin and Tysabri scFv were produced with and without CyDisCo, while the other seven antibody fragments were produced only with CyDisCo present to catalyse native disulfide bond formation. The proteins were purified by IMAC and the quality analysed by SDS–PAGE. Similar patterns were observed as observed in DWP expression (see Fig. 3a as example). The purified proteins were quantified using absorbance at 280 nm (Table 2). While the yields of scFv were in line with estimates from SDS–PAGE of lysates, the yields of the Fab fragments were on average two-fold lower than expected, suggesting that the standard IMAC protocol or the placement of the hexahistidine tag at the end of the heavy chain was non-optimal. The yields in mg/L obtained for the nine antibody fragments tested in shake flask scale were on average 103 % of those obtained in DWP (compare Table 1 with Table 2) demonstrating the ease of going from screening scale to a scale suitable for structural or functional studies using CyDisCo in EnPresso B media.

**Table 2:** Yields of antibody fragments purified from shake flask cultures

Fragment type	Antibody name	Purified yield
scFv	Herceptin (human IgG_1_)	251 mg/L (with CyDisCo)
		167 mg/L (without CyDisCo)
	Tysabri (human IgG_4_)	169 mg/L (with CyDisCo)
		141 mg/L (without CyDisCo)
	3211 (mouse IgG_1_)	11 mg/L
	3M8O (human IgA_1_)	68 mg/L
	2R56 (human IgE)	32 mg/L
Fab	Maa48 (human IgG_1_)	42 mg/L
	3211 (mouse IgG_1_)	13 mg/L
	3M8O (human IgA_1_)	19 mg/L
	1QLR (human IgM)	11 mg/L

Yields were determined by A280 measurements. Extinction coefficients used were calculated using ProtParam and assuming all of the cysteines present are in disulfide bonds. Except where stated all fragments were made in the presence of CyDisCo components

To confirm that the proteins obtained were correctly folded far UV circular dichroism (CD) was performed. All of the proteins showed a CD spectra with a minima around 217 nm (Fig. 3b), consistent with the β-sheet found in the Ig fold.

To further confirm that the antibody fragments obtained were natively folded, a selection of them were tested for their ability to bind to their reported antigens. The scFv and Fab of 3211 bound to a peptide fragment of BNP, the antigen it was raised against (Fig. 3c). Due to the availability of suitable substrates, the binding specificity of the Fab fragment of Maa48 was analysed in more detail. Maa48 Fab bound to malondialdehyde-acetaldehyde (MAA)-modified antigen, but not to malondialdehyde (MDA)-modified antigen or to the non-modified antigen (Fig. 3d, e). In addition, Maa48 Fab did not bind to copper oxidized LDL or carbamylated LDL. These results match the published specificity of Maa48 (also known as Fab-pre; [14]) and imply that antibody fragments produced using CyDisCo in the cytoplasm of *E. coli* retain biological activity and specificity.

### Analysis of Herceptin and Tysabri scFv

Both Herceptin and Tysabri were unusual in that the scFv were solubly expressed in high yields in the absence of CyDisCo components i.e. under conditions in which disulfide bond formation should not occur. While the purified yields in the absence of CyDisCo were up to one-third lower of that obtained in the presence of CyDisCo components, the high level produced suggests either that the proteins are soluble and proteolytically stable in the absence of disulfide bond formation or that disufide bond formation occurs via an alternative route for these scFv. To examine the redox state of these the IMAC purified scFv produced in the presence and absence of CyDisCo were analysed.

Herceptin scFv produced using CyDisCo showed a small mobility shift in SDS–PAGE in the presence and absence of β-mercaptoethanol (data not shown), indicating that it contained at least one intra-molecular disulfide bond. In contrast the same scFv produced in the absence of CyDisCo showed no mobility shift. The absence of disulfide bonds in both Herceptin and Tysabri scFv when produced in the absence of CyDisCo was confirmed by electrospray mass spectrometry (Table 3). Similarly both scFv were confirmed to contain two disulfide bonds when made in the presence of CyDisCo.

**Table 3:** Molecular mass of scFv fragments produced with and without CyDisCo

scFv	CyDisCo	NEM treatment	Molecular mass (Da)
Herceptin	−	−	27188
	−	+	27688
	+	−	27185
	+	+	27185
Tysabri	−	−	27753
	−	+	28256
	+	−	27751
	+	+	27752

Masses were determined by electrospray mass spectrometry with (+) or without (−) prior treatment with N-ethylmaleimide (*NEM*) which reacts with free thiol groups. Reaction with NEM increases the mass of the protein by 125 Da. In the absence of CyDisCo a 500 Da shift in mass was observed upon NEM treatment consistent with four free thiol groups i.e. no disulfide bonds. In contrast in the presence of CyDisCo no shift in mass was observed upon NEM treatment consistent with both disulfide bonds being formed

## Conclusions

By using the CyDisCo system for disulfide bond formation we were able to generate high yields of folded, biologically active, antibody fragments (scFv and Fab) in the cytoplasm of *E. coli* with a >90 % success rate. The direct, systematic, side by side comparison of eleven different antibodies of eight different types with no protein dependent optimization demonstrates the flexibility of the system. The use of CyDisCo for the production of proteins containing up to nine disulfide bonds [10], suggests that it could also be used to produce antibody fragments with engineered disulfide bonds to increase stability [29].

Of the 22 constructs tested only two, the scFv of Herceptin and Tysabri, were produced in a soluble state in the absence of CyDisCo, the rest being produced as inclusion bodies. Sequence analysis of the eleven scFv (see Additional file 1 for an alignment) showed no consensus in sequence either at a global or at a local level to explain why these two alone were able to fold to a soluble stable state in the absence of disulfide bond formation. Similarly no consensus was observed when comparing the available structures of the variable domains of these two antibodies in comparison with those antibodies whose scFv were only produced in a soluble state when CyDisCo components were present.

In vitro studies of antibody folding strongly suggest the involvement of multiple protein folding factors beyond those catalysing disulfide bond formation (reviewed in [30, 31]). In particular *cis*-prolyl isomerization is a rate limiting step that requires the action of a peptidyl prolyl *cis*–*trans* isomerase (PPI) and the first constant domain of the heavy chain (C_H_1) requires the action of the molecular chaperone BiP, a HSP70 family member, to retain it in a folding competent state until the heavy and light chain associate. Our system adds neither of these factors and yet yields of purified protein of up to 250 mg/L of folded scFv and 42 mg/L of folded Fab are obtained from shake flasks without protein specific optimization. This suggests that intrinsic *E. coli* proteins fulfil these roles. *E. coli* has six cytoplasmic PPIs (the gene products of *fkpB*, *fkbX*, *ppiB*, *ppiC*, *slyD* and *tig*) and one cytoplasmic HSP70 family member (the gene product of *dnaK*) and the most probably explanation is that these fulfil the roles necessary for antibody fragment folding. Only the catalysts of native disulfide bond formation need to be added.

The production levels of the scFv and Fab obtained varied by circa 60-fold for the 10 scFv and nearly 20-fold for the 10 Fab produced. No patterns were observed based on antibody subtype—for example the best produced scFv were IgG_1_ and IgG_4_ subtypes, while the best and worse Fab were both IgG_1_ subtypes. In addition, no patterns were observed based on sequence analysis (see Additional file 1: Figures S1 and S2) or on structural analysis—based on the available structures for eight of the antibody fragments. In general the levels of Fabs were lower than those of scFv (23 mg/L on average compared with. 63 mg/L on average, but note the bias caused by the two scFv which do not have a requirement for CyDisCo). However, there was no simple correlation between the production levels of the scFv and that of the corresponding Fab, even within a single antibody subtype. This is neatly exemplified by Humira and Maa48, both human IgG_1_ subtypes, with the yields from DWP of the Fab compared with the scFv being circa 6× lower for Humira, but circa 10× higher for Maa48 (Table 1).

As we wanted to have a systematic side-by-side comparison of the abilities of the system, no systematic protein specific optimization was performed. As such this makes direct comparison with published data for other production systems problematic as usually protein specific optimization is performed as there only one or two target proteins rather than, as here, a wider ranging proof of concept for a class of proteins. Other systems, such as expression in the endoplasmic reticulum of CHO or yeast or periplasmic expression in *E. coli* have achieved yields in excess of 1 g/L for antibodies and/or antibody fragments after optimization. Preliminary data using CyDisCo suggests that with optimization higher yields may be obtained, with an increase in yield of at least twofold having already been obtained for more than half of the 22 constructs tested here. For example yields of more than 150 mg/L of Maa48 Fab and more than 100 mg/L of 2R56 scFv have been obtained from DWP expression during preliminary optimization. As per any heterologous protein expression such optimization may need to include choice of vector, codon usage, translation initiation, mRNA stability, relative expression levels of subunits in multi-subunit complexes, bacterial strain, media and expression, induction and purification conditions, etc. Further studies aimed towards increasing yields and being able to make sequence based predictions of yields are ongoing. However, preliminary data from these antibody fragments, combined with data from successful expression of more than 100 other proteins using CyDisCo, within our research group suggests that one of two effects may be limiting yields for specific antibody fragments: (1) proteolytic stability i.e. that the protein is made, folds, but the folded state is prone to proteolysis by cytoplasmic proteases; (2) solubility of the folding intermediates or, less commonly, of the final folded state. While the solubility of the final native state can be readily determined, or to some extent predicted based on sequence, the solubility of folding intermediates is currently unpredictable. However, testing a wider range of scFv to identify more examples like Herceptin and Tysabri derived scFv which are able to reach a stable, soluble state in the absence of disulfide bond formation may allow further elucidation of factors which increase the solubility of folding intermediates of antibody fragments and hence increase yields. Such data may also impact on yields obtained in other production systems. To date no patterns have been identified to allow prediction of which factors require optimization for any given protein, except that disruption of the reducing pathways e.g. the use of a *ΔtrxB*/*Δgor* strain such as rosetta-gami, combined with CyDisCo components expressed at high levels (such as from the plasmid used here) is usually deleterious to the production of native disulfide bonds. This effect probably arises as the system becomes over-oxidizing and is unable to catalyse isomerization of non-native disulfides to the native state.

To date CyDisCo works in all *E. coli* strains tested and in all media tested, including minimal media in batch or batch-fed fermentation (manuscript in preparation) and no deleterious effects have been observed of CyDisCo component expression in production strains in any media tested. Hence, despite the requirement for further optimization and scale up our results opens up a wide range of possibilities for the production of therapeutic and diagnostic proteins on both a laboratory and industrial scale.

## Methods

### Vector construction

Expression vectors (see Table 4 for vectors used in this study) were made by standard molecular biology techniques.

**Table 4:** Details of the plasmid vectors used in this study

Name	Type	scFv	Fab
Humira	IgG_1_ human	pJV77	pJV59
Maa48	IgG_1_ human	pJV79	pJV11
K2	IgG_1_ human	pJV80	pJV12
Avastin	IgG_1_ humanized	pJV76	pYU177
Herceptin	IgG_1_ humanized	pJV78	pJV60
Tysabri	IgG_4_ humanized	pJV81	pJV61
3211	IgG_1_ mouse	pJV82	pJV13
Mab123	IgG_2_ mouse	pJV83	pYU183
3M8O	IgA_1_ human	pJV84	pJV62
2R56	IgE human	pJV85	pJV63
1QLR	IgM human	pJV86	pJV64
CyDisCo components	Erv1p + PDI	pMJS205	
CyDisCo negative control	None	pAG82	

Genes for the Erv1p, mature PDI along with the heavy and light chains of the antibodies tested (lacking the N-terminal signal sequence) were synthesized codon optimized for *E .coli* expression (GenScript; Additional file 1: Figure S3). IgE and IgM heavy chains were synthesized without the C-terminal region involved in oligomerization.

The expression vector used was a modified version of pET23 in which the T7 promoter was replaced with Ptac promoter from previously modified (SpeI site inserted) pMal-p2X [10] by digesting the pMal-p2X with MscI/SpeI and ligating the fragment into MscI/XbaI digested pET23. Synthetic multi-cloning sites for Fab fragments (EcoRV/XhoI) and scFv fragments (EcoRV/CelII) were synthesised (GenScript) and ligated into this vector backbone.

**Fig. 4:**
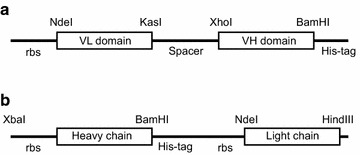
Structure of the expression vectors used in this study. **a** scFv vector. The spacer region including the KasI and XhoI sites encodes for the sequence -Gly-Ala-Ser-(Gly_4_-Ser)_3_-Ser- while the hexahistidine-tag including the BamHI site adds Gly-Ser-His_6_. *rbs* = ribsome binding site. The initiating Met is included in the NdeI site (CATATG); **b** Fab vector. This polycistronic vector includes two ribosome binding sites (*rbs*) to initiate translation of the heavy and light chain. The hexahistdine-tag including the BamHI site adds Gly-Ser-His_6_

The variable domains of the light and heavy chain were amplified by PCR from the synthetic genes and cloned into a synthetic multi-cloning site using NdeI/KasI (light chain) and XhoI/BamHI (heavy chain) to generate a scFv with a C-terminal hexahistidine-tag (Fig. 4). Since the vector backbone, tag and linker region were constant any differences in scFv production comes from the variable regions.

The truncated heavy chain for Fab production was amplified by PCR from the synthetic gene and cloned XbaI/BamHI into a polycistronic vector with the light chain (cloned NdeI/Hind III). These polycistronic vectors include two ribosome binding sites (rbs) and make two proteins by co-expression from two translation initiation sites (Fig. 4). All heavy chain fragments included a C-terminal hexahistidine-tag. The tag was placed on the heavy chain rather than the light chain as preliminary studies on other Fab fragments had suggested that in some cases soluble light chain could be generated and purified in the absence of heavy chain co-expression whereas the opposite was not observed i.e. having the tag on the heavy chain was designed to increase the quality of the final product.

A polycistronic expression construct for codon optimized Erv1p and codon optimized mature PDI was made in modified pET23 as described previously [9]. The polycistronic fragment was transferred into the new vector with Ptac promoter by cloning XbaI/XhoI. From there the fragment containing Ptac promoter, codon optimized Erv1p and codon optimized PDI was cloned NsiI/AvrII into modified a pLysSBAD-vector described previously [10] to generate pMJS205 expression vector. A control construct identical to pMJS205, except lacking the genes for Erv1p and PDI was made by removing the genes by NdeI/SpeI digestion and ligating in short annealed primers with complementary sticky ends.

All plasmid purification was performed using the Gen-Elute HP Plasmid Miniprep Kit (Sigma Aldrich) and all purification from agarose gels was performed using the Gel/PCR DNA Fragments Extraction Kit GeneAid), both according to the manufacturers’ instructions.

All plasmids generated were sequenced to ensure there were no errors in the cloned genes.

### Protein expression

For expression in EnPresso B media in 24 deep well plates, *E. coli* strains containing expression vectors were streaked out from glycerol stocks stored at −70 °C onto LB agar plates containing 5 g/L glucose and suitable antibiotics to allow for selection (100 μg/ml ampicillin for pET23 derivatives, 35 μg/ml chloramphenicol for pLysS derivatives) and the plates incubated at 37 °C overnight. The next day one–three colonies from these plates were used to inoculate 2 ml of LB media supplemented with 2 g/L glucose, containing suitable antibiotics, and the cultures grown at 30 °C, 200 rpm (2.5 cm radius of gyration) in 24 deep well plates covered with an oxygen permeable membrane for 6–8 h. These cultures were used to seed 24 deep well plates containing 3 mls of EnPresso B media (Biosilta Oy; as manufacturer’s instructions) per well containing suitable antibiotics and the cultures grown at 30 °C, 200 rpm (5 cm radius of gyration) in 24 deep well plates covered with an oxygen permeable membrane for approximately 16 h. The cultures were then boosted (as manufacturer’s instructions) and induced with 0.5 mM IPTG. Cultures were harvested after a further 24 h of growth. Final OD_600_ values of the cultures were in the range 20–37. The cells were collected by centrifugation and resuspended in 3 mls of 50 mM sodium phosphate pH 7.4, 20 μg/ml DNase, 0.1 mg/ml egg white lysozyme. After 10 min incubation the resuspended cultures were frozen. Cells were lysed by freeze-thawing.

Protein expression in shake flasks was as per 24 deep well plates except the media volume was 25 mls (250 ml flask) of EnPresso B media and cultures were grown at 30 °C, 250 rpm (2.5 cm radius of gyration). Resuspension was done in the same volume as the initial culture.

### Protein purification and analysis

Purification of hexa-histidine tagged proteins was performed by standard immobilized metal affinity chromatography using HisPur Cobalt Superflow Agarose (Thermos Scientific) resin under native conditions following clearance of the cell lysate by centrifugation (4000 rpm, 20 min, 4 °C) for 24 deep well plate. For 3 ml cultures from 24 deep well plates IMAC was performed using 0.5 ml resin in small gravity feed columns. The resin was washed with 2 × 5 mls of water, equilibrated with 2 × 5 mls of 50 mM phosphate buffer (pH 7.4). After loading the sample the column was equilibrated with 5 ml of 50 mM phosphate buffer (pH 7.4), washed with 4 × 5 mls of wash buffer (50 mM sodium phosphate, 5 mM imidazole, 0.3 M sodium chloride; pH 7.4) then 5 mls of 50 mM sodium phosphate (pH 7.4) before elution with 3 × 0.7 mls of 50 mM sodium phosphate, 150 mM imidazole (pH 7.4). For 25 ml cultures the same protocol was used with the following changes: 1.0 ml of resin; 6 × 5 mls of wash buffer; elution with 4 × 1 ml of buffer. Where needed, 2.5 ml of eluted sample was desalted into 50 mM sodium phosphate (pH 7.4) on PD-10 columns (GE Healthcare). Appropriate samples were treated with 20 mM NEM for 20 min at room temperature prior to making SDS–PAGE samples or mass spectrometry analysis.

### Protein analysis

Far-UV circular dichroism spectra were recorded on a Chirascan- plus CD spectrophotometer. All scans were collected at 25 °C as an average of four scans, using a cell with a path length of 0.1 cm, scan speed 2 nm/s, step size 0.5 nm, a spectral band width of 1.0 nm. The maximal HT voltage was 750 V.

For determining binding of 3211 Fab and scFv to their ligand, 100 ng of recombinant NTproBNP1-76 [32] in 0.1 M sodium bicarbonate buffer pH 9.6 was coated per well on a shaking ELISA plate over-night at 4 °C. The wells were emptied and rinsed three times with 250 μl of 1xPBS (20 mM phosphate, 150 mM sodium chloride, pH 7.4) containing 0.05 %v/v tween 20 and then incubated with 250 μl of blocking buffer (0.2 % gelatin, 0.5 % BSA, 0.05 % v/v tween20 in 1xPBS, pH 7.4) for 20 min at room temperature. Then 100 μl samples containing 0–20 ng of scFv or Fab, diluted in the blocking buffer were incubated for 2 h at room temperature shaking. After removal of the sample and washing the wells six times with 300 μl of 1xPBS containing 0.05 % v/v tween 20, 100 μl of alkaline phosphatase labelled anti-HIS antibody (Sigma) diluted 1:10000 in blocking buffer was added and the reactions incubated for 1 h at room temperature. After removal of the detection antibody and washing the wells six times with 300 μl of 1xPBS containing 0.05 % tween 20, the 1 mg/ml pNPP-substrate solution (Sigma) in 0.2 M Tris, 5 mM MgCl_2_ was added and incubated for 30 min at room temperature. The absorbance at 405 nm was measured with a Tecan Infinite M1000PRO multilabel reader.

For determining the specificity of Maa48 Fab binding, the ligand (MAA-LDL, MDA-LDL, copper oxidized-LDL, carbamylated-LDL, native LDL, MAA-BSA, MDA-BSA or native BSA; sources) at 0–20 μg/ml concentration in PBS was bound to an ELISA plate at 4 °C overnight. The antigens were prepared as described [15]. The plate was washed three times with 0.27 mM EDTA in PBS using an automated plate washer. Nonspecific binding was blocked with 0.5 % fish gelatin and 0.27 mM EDTA in PBS for 1 h at room temperature. Maa48 Fab (1 µg/ml) was incubated for 1 h at room temperature. Alkaline phosphatase-conjugated anti-human IgG (Fab) (Sigma) was used as a secondary antibody and LumiPhos 530 (Lumigen) as a substrate in the assay [14]. The chemiluminescence was measured as relative light units (RLU) with a Wallac Victor3 multilabel reader (Perkin Elmer).

Masses of purified and desalted proteins, treated and not treated with 20 mM NEM were measured by LCMS with an Aquity UPLC-system (Waters) connected to a Synapt G1 Q-ToF –type mass spectrometer. The analytical column was a BEH 300 C4, 2.1 ×100 mm (Waters) run at 0.4 ml/min using a gradient from 3 % acetonitrile in water/0.1 % formic acid to 70 % acetonitrile over 15 min. Samples were acidified with trifluoracetic acid to about 0.5 % v/v and 5 µl of the sample was injected. The mass spectrometer was operated in sensitivity mode with lock mass corrected 1 s scans in continuous mode for m/z 400–2000. Capillary voltage was 3.5 kV, cone voltage 30 V. Mass spectra were base line subtracted and deconvoluted with MaxEnt1.

## Additional file

10.1186/s12934-016-0419-5 scFv alignment. **Figure S2.** Phylograms. **Figure S3.** Nucleotide sequences.

## Abbreviations

DWP: deep well plate
NEM: N-ethyl maleimide
PDI: protein disulfide isomerase
