# Supplementary material for: Systematic screening of soluble expression of antibody fragments in the cytoplasm of E. coli
Source: Microb Cell Fact. 2016 Jan 25;15:22. doi: 10.1186/s12934-016-0419-5 (PMC4727284; doi:10.1186/s12934-016-0419-5)
Supplement: Supplementary file 1 — 10.1186/s12934-016-0419-5 scFv alignment. Figure S2. Phylograms. Figure S3. Nucleotide sequences. [file 12934_2016_419_MOESM1_ESM.pdf]

**Additional Figure 1: scFv Alignment**

An alignment of eight of the scFv examined using Clustal Omega. The complementary determining regions (CDRs) are underlined. The sequences of the three antibodies with potential diagnostic use are not included.

```
1QLR           MEIVLTQSPATLSLSPGERATLSCGASQSV----SSNYLAWYQQKPGQAPRLLIYDASSR
3M8O           MDIVMTQSPLSLSVTPGEPASISCRSSQSLLRRDGHNDLEWYLQKPGQSPQPLIYLGSTR
Tysabri        MDIQMTQSPSSLSASVGDRVTITCKTSQDI-----NKYMAWYQQTPGKAPELLIHYTSAL
Mab123         MDIVLTQSPSSLSASLGDTITITCHASQNI-----NVWLSWYQQKPGNIPKLLIYKASNL
2R56           MDIVMTQSPSSLSASVGDRVTITCRASQGI-----SSRLAWYQQKPGKAPKLLIYAASSL
Avastin        MDIQMTQSPSSLSASVGDRVTITCSASQDI-----SNYLNWYQQKPGKAPKVLIYFTSSL
Humira         MDIQMTQSPSSLSASVGDRVTITCRASQGI-----RNYLAWYQQKPGKAPKLLIYAASTL
Herceptin      MDIQMTQSPSSLSASVGDRVTITCRASQDV-----NTAVAWYQQKPGKAPKLLIYSASFL
               *:* :**** :** : *:  :::* :** :        : ** *.**: *. **:  *

1QLR           ATGIPDRFSGSGSGTDFTLTISRLEPEDFAVYYCQQYGSSPLTFGGGTKVEIKGASGGGG
3M8O           ASGVPDRFSGSGSGTDFTLKIIRVEAEDAGTYYCMQNKQTPLTFGQGTRLEIKGASGGGG
Tysabri        QPGIPSRFSGSGSGRDYTFTISSLQPEDIATYYCLQYDN-LWTFGQGTKVEIKGASGGGG
Mab123         HTGVPSRFSGSGSGTGFTLTISSLQPEDIATYYCQQGQSYPLTFGGGTKLEIKGASGGGG
2R56           QSGVPSRFSGSGSGTEFTLTISSLQPEDFATYYCQQYHSYPWTFGQGTKLEIKGASGGGG
Avastin        HSGVPSRFSGSGSGTDFTLTISSLQPEDFATYYCQQYSTVPWTFGQGTKVEIKGASGGGG
Humira         QSGVPSRFSGSGSGTDFTLTISSLQPEDVATYYCQRYNRAPYTFGQGTKVEIKGASGGGG
Herceptin      YSGVPSRFSGSRSGTDFTLTISSLQPEDFATYYCQQHYTTPPTFGQGTKVEIKGASGGGG
                 *:*.***** **  :*:.*  :: ** ..*** :      *** **::**********

1QLR           SGGGGSGGGGSSEVQLQQWGAGLLKPSETLSLTCAVYGGSFSDYYWSWIRQPPGKGLEWI
3M8O           SGGGGSGGGGSSEVQLVESGGGLVQPGGSLKLSCAASGFTLSGSNVHWVRQASGKGLEWV
Tysabri        SGGGGSGGGGSSQVQLVQSGAEVKKPGASVKVSCKASGFNIKDTYIHWVRQAPGQRLEWM
Mab123         SGGGGSGGGGSSEVKLQESGGGLVQPGGSLKLSCATSGFTFSDYYMYWVRQTPEKRLEWV
2R56           SGGGGSGGGGSSQVSLRESGGGLVQPGRSLRLSCTASGFTFRHHGMTWVRQAPGKGLEWV
Avastin        SGGGGSGGGGSSEVQLVESGGGLVQPGGSLRLSCAASGYTFTNYGMNWVRQAPGKGLEWV
Humira         SGGGGSGGGGSSEVQLVESGGGLVQPGRSLRLSCAASGFTFDDYAMHWVRQAPGKGLEWV
Herceptin      SGGGGSGGGGSSEVQLVESGGGLVQPGGSLRLSCAASGFNIKDTYIHWVRQAPGKGLEWV
               ************:*.* : *. : :*. :: ::* . * .:      *:**   : ***:

1QLR           GEINHS---GSTNYNPSLKSRVTISVDTSKNQFSLKLSSVTAADTAVYYCARPPHDT--S
3M8O           GRIKRNAESDATAYAASMRGRLTISRDDSKNTAFLQMNSLKSDDTAMYYCVIRGDV----
Tysabri        GRIDPA--NGYTKYDPKFQGRVTITADTSASTAYMELSSLRSEDTAVYYCAREGYYGNYG
Mab123         AYISNG--GGSTYYPDTVKGRFTISRDNAKNTLYLQMSRLKSEDTAMYYCARHGGY----
2R56           ASLSGS--GTKTHFADSVKGRFTISRDNSNNTLYLQMDNVRDEDTAIYYCAKAKRV--GA
Avastin        GWINTY--TGEPTYAADFKRRFTFSLDTSKSTAYLQMNSLRAEDTAVYYCAKYPHYYGSS
Humira         SAITWN--SGHIDYADSVEGRFTISRDNAKNSLYLQMNSLRAEDTAVYYCAKVSYL--ST
Herceptin      ARIYPT--NGYTRYADSVKGRFTISADTSKNTAYLQMNSLRAEDTAVYYCSRWGGD---G
               . :          :   .. *.*:: * : .   :::. :   ***:***

1QLR           GHYWNYWGQGTLVTVSSGSHHHHHH
3M8O           --YNRQWGQGTLVTVSSGSHHHHHH
Tysabri        VYAMDYWGQGTLVTVSSGSHHHHHH
Mab123         -YAMDYWGQGTTVTVSSGSHHHHHH
2R56           TGYFDLWGRGTLVTVSSGSHHHHHH
Avastin        HWYFDVWGQGTLVTVSSGSHHHHHH
Humira         ASSLDYWGQGTLVTVSSGSHHHHHH
Herceptin      FYAMDYWGQGTLVTVSSGSHHHHHH
                     **:** *************
```

**Additional Figure 2: Phylograms**
Phylograms of the VH (panel A) and VL (panel B) of the eleven antibodies examined. Phylograms were generated using Clustal Omega. No correlation can be observed with yields obtained.

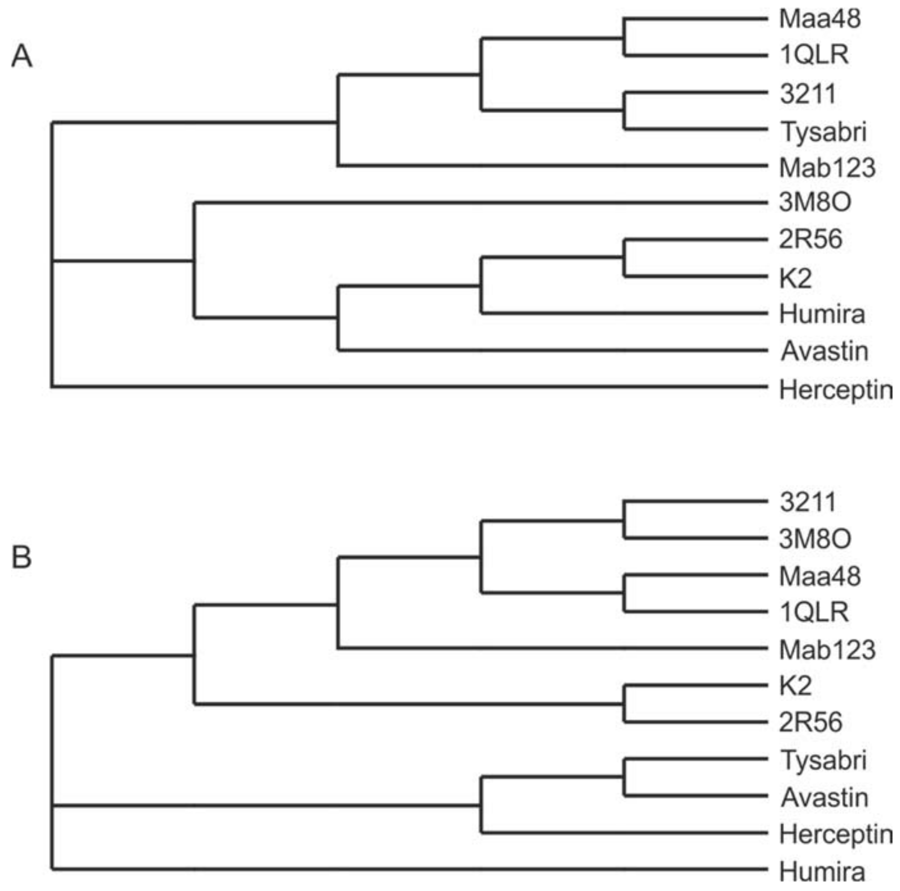

**Additional Figure 3: Nucleotide sequences**

Codon optimized nucleotide sequences of eight of the antibodies examined. For each antibody the scFv, and Fab heavy and light chains are shown. The sequences of the three antibodies with potential diagnostic use are not included.

Humira scFv
ATGGACATCCAAATGACCCAATCCCCGTCCAGTCTGTCCGCTTCAGTCGGTGACCGTGTGACCA
TTACGTGCCGTGCCAGCCAAGGTATCCGTAACTATCTGGCGTGGTACCAGCAAAAACCGGGTA
AAGCCCCGAAACTGCTGATTTATGCCGCCTCCACCCTGCAGTCAGGCGTGCCGTCGCGCTTTAG
CGGCTCTGGTAGTGGCACGGATTTCACCCTGACGATCAGCTCTCTGCAGCCGGAAGACGTTGCG
ACCTATTACTGCCAACGTTATAATCGTGCGCCGTACACCTTTGGTCAGGGCACGAAAGTCGAAA
TTAAAGGCGCCAGCGGCGGTGGCGGTTCTGGTGGCGGTGGCAGCGGTGGCGGTGGCTCGAGCG
AAGTCCAACTGGTCGAATCGGGCGGTGGTCTGGTTCAGCCGGGTCGTTCCCTGCGTCTGTCGTG
TGCTGCCTCGGGTTTTACGTTTGATGACTATGCAATGCATTGGGTCCGTCAGGCTCCGGGCAAA
GGTCTGGAATGGGTGTCTGCGATTACCTGGAACAGTGGCCACATCGATTATGCCGACTCTGTGG
AAGGTCGTTTTACGATTTCGCGCGATAACGCGAAAAATAGCCTGTACCTGCAGATGAATTCTCT
GCGCGCGGAAGACACCGCCGTTTATTACTGCGCGAAAGTCTCGTATCTGAGCACGGCCAGCTCT
CTGGATTACTGGGGCCAGGGTACCCTGGTTACCGTCAGTTCCGGATCCCATCACCATCACCATC
ACTAA

Humira Fab heavy chain
ATGGAAGTCCAACTGGTCGAA
TCGGGCGGTGGTCTGGTTCAGCCGGGTCGTTCCCTGCGTCTGTCGTGTGCTGCCTCGGGTTTTAC
GTTTGATGACTATGCAATGCATTGGGTCCGTCAGGCTCCGGGCAAAGGTCTGGAATGGGTGTCT
GCGATTACCTGGAACAGTGGCCACATCGATTATGCCGACTCTGTGGAAGGTCGTTTTACGATTT
CGCGCGATAACGCGAAAAATAGCCTGTACCTGCAGATGAATTCTCTGCGCGCGGAAGACACCG
CCGTTTATTACTGCGCGAAAGTCTCGTATCTGAGCACGGCCAGCTCTCTGGATTACTGGGGCCA
GGGTACCCTGGTTACCGTCAGTTCCGCTAGCACCAAAGGCCCGAGCGTTTTCCCGCTGGCTCCG
TCATCGAAATCTACCAGTGGCGGTACCGCCGCACTGGGTTGTCTGGTGAAAGATTATTTTCCGG
AACCGGTGACCGTTTCTTGGAACAGTGGCGCACTGACCAGCGGTGTGCATACGTTCCCGGCTGT
TCTGCAGAGCTCTGGCCTGTATAGCCTGAGTTCCGTGGTTACCGTTCCGTCATCGAGCCTGGGT
ACCCAAACGTACATTTGCAACGTCAATCATAAACCGAGTAATACCAAAGTGGATAAGAAAGTG
GAACCGAAATCCTGTGACAAAACCGGATCCCATCACCATCACCATCACTAA

Humira Fab light chain
ATGGACATCCAAATGACCCAATCCCCGTCCAGTCTGTCCGCTTCAGTCGGTGACCGTGTGACCA
TTACGTGCCGTGCCAGCCAAGGTATCCGTAACTATCTGGCGTGGTACCAGCAAAAACCGGGTA
AAGCCCCGAAACTGCTGATTTATGCCGCCTCCACCCTGCAGTCAGGCGTGCCGTCGCGCTTTAG
CGGCTCTGGTAGTGGCACGGATTTCACCCTGACGATCAGCTCTCTGCAGCCGGAAGACGTTGCG
ACCTATTACTGCCAACGTTATAATCGTGCGCCGTACACCTTTGGTCAGGGCACGAAAGTCGAAA
TTAAACGTACCGTTGCGGCGCCGTCGGTCTTTATCTTCCCGCCGAGCGATGAACAACTGAAATC
TGGTACCGCGAGTGTGGTTTGTCTGCTGAACAATTTTTATCCGCGTGAAGCGAAAGTCCAGTGG
AAGGTGGACAACGCCCTGCAGTCTGGCAATAGTCAAGAATCCGTGACCGAACAAGATTCAAAA
GACTCGACGTACAGCCTGAGTTCCACCCTGACGCTGAGCAAGGCAGATTATGAAAAACATAAG
GTGTACGCTTGCGAAGTTACCCACCAGGGTCTGTCCCTGCCGGTTACGAAATCATTCAACCGCG
GCGAATGTTAA

Avastin scFv
ATGGATATTCAAATGACCCAGAGCCCGTCCAGCCTGTCGGCGTCCGTTGGTGACCGTGTTACGA
TTACCTGCAGTGCATCCCAGGATATTAGCAACTATCTGAATTGGTACCAGCAAAAACCGGGCAA
GGCTCCGAAAGTTCTGATCTATTTTACCAGCTCTCTGCATTCTGGCGTCCCGAGTCGTTTTTCAG
GCTCGGGTAGCGGCACCGATTTCACCCTGACGATTAGTTCCCTGCAGCCGGAAGACTTTGCAAC
GTATTACTGCCAGCAATACAGCACCGTCCCGTGGACGTTCGGTCAAGGCACCAAGGTGGAAAT

TAAAGGCGCCAGCGGCGGTGGCGGTTCTGGTGGCGGTGGCAGCGGTGGCGGTGGCTCGAGCGA
AGTCCAACTGGTCGAATCGGGTGGTGGTCTGGTCCAACCGGGTGGCAGTCTGCGTCTGTCCTGC
GCGGCCTCAGGCTATACCTTTACGAACTACGGTATGAATTGGGTGCGTCAGGCACCGGGTAAA
GGTCTGGAATGGGTTGGCTGGATTAACACCTATACGGGTGAACCGACCTACGCAGCTGATTTCA
AACGTCGCTTTACGTTCAGCCTGGACACCTCTAAAAGTACGGCATATCTGCAGATGAACAGCCT
GCGTGCGGAAGATACCGCCGTTTATTACTGCGCTAAATATCCGCATTATTACGGTAGCTCTCAC
TGGTACTTTGACGTGTGGGGCCAGGGTACCCTGGTTACCGTCAGTTCCGGATCCCATCACCATC
ACCATCACTAA

Avastin Fab heavy chain
ATGGAAGTCCAACTGGTCGAATCGGGTGGTGGTCTGGTCCAACCGGGTGGCAGTCTGCGTCTGT
CCTGCGCGGCCTCAGGCTATACCTTTACGAACTACGGTATGAATTGGGTGCGTCAGGCACCGGG
TAAAGGTCTGGAATGGGTTGGCTGGATTAACACCTATACGGGTGAACCGACCTACGCAGCTGA
TTTCAAACGTCGCTTTACGTTCAGCCTGGACACCTCTAAAAGTACGGCATATCTGCAGATGAAC
AGCCTGCGTGCGGAAGATACCGCCGTTTATTACTGCGCTAAATATCCGCATTATTACGGTAGCT
CTCACTGGTACTTTGACGTGTGGGGCCAGGGTACCCTGGTTACCGTCAGTTCCGCGAGCACCAA
AGGTCCGAGCGTTTTCCCGCTGGCACCGTCATCGAAATCCACCTCAGGCGGTACGGCAGCACTG
GGTTGTCTGGTGAAAGATTATTTTCCGGAACCGGTGACCGTTTCATGGAACTCGGGCGCACTGA
CCAGCGGTGTTCATACGTTCCCGGCTGTCCTGCAGAGCTCTGGCCTGTATAGCCTGAGTTCCGT
GGTTACCGTCCCGTCATCGAGCCTGGGTACCCAAACGTACATTTGCAACGTGAATCATAAACCG
TCCAATACGAAGGTGGATAAAAAGGTTGAACCGAAATCATGTCATCACCATCACCATCACTAA

Avastin Fab light chain
ATGGATATTCAAATGACCCAGAGCCCGTCCAGCCTGTCGGCGTCCGTTGGTGACCGTGTTACGA
TTACCTGCAGTGCATCCCAGGATATTAGCAACTATCTGAATTGGTACCAGCAAAAACCGGGCAA
GGCTCCGAAAGTTCTGATCTATTTTACCAGCTCTCTGCATTCTGGCGTCCCGAGTCGTTTTTCAG
GCTCGGGTAGCGGCACCGATTTCACCCTGACGATTAGTTCCCTGCAGCCGGAAGACTTTGCAAC
GTATTACTGCCAGCAATACAGCACCGTCCCGTGGACGTTCGGTCAAGGCACCAAGGTGGAAAT
TAAACGTACGGTTGCGGCCCCGTCGGTCTTTATCTTCCCGCCGTCAGATGAACAGCTGAAATCC
GGTACCGCCTCAGTGGTTTGTCTGCTGAACAATTTTTATCCGCGCGAAGCAAAGGTCCAATGGA
AAGTGGACAACGCTCTGCAGTCTGGCAATAGTCAAGAATCCGTGACCGAAGAAGATTCAAAGG
ACTCGACGTACAGCCTGTCATCGACCCTGACGCTGTCTAAAGCGGATTATGAAAAACATAAGGT
GTACGCCTGCGAAGTTACCCACCAGGGTCTGAGCTCTCCGGTTACGAAAAGTTTCAACCGCGGC
GAATGTTAA

Herceptin scFv
ATGGATATTCAGATGACCCAGAGCCCGTCCAGCCTGTCAGCAAGCGTTGGCGACCGTGTTACGA
TTACCTGCCGTGCCTCGCAAGATGTGAACACCGCGGTGGCCTGGTATCAGCAAAAACCGGGCA
AAGCACCGAAACTGCTGATTTATAGTGCTTCCTTTCTGTACTCTGGTGTTCCGTCCCGTTTCTCA
GGCTCGCGCAGCGGTACCGATTTTACCCTGACGATCAGCTCTCTGCAGCCGGAAGACTTCGCCA
CGTATTACTGCCAGCAACACTACACGACCCCGCCGACCTTCGGCCAGGGCACCAAAGTGGAAA
TCAAAGGCGCCAGCGGCGGTGGCGGTTCTGGTGGCGGTGGCAGCGGTGGCGGTGGCTCGAGCG
AAGTCCAACTGGTCGAATCGGGTGGTGGTCTGGTTCAGCCGGGCGGTTCACTGCGTCTGTCGTG
TGCTGCCTCGGGTTTCAACATCAAAGATACCTATATTCATTGGGTGCGTCAGGCCCCGGGTAAA
GGTCTGGAATGGGTTGCCCGCATCTACCCGACCAACGGCTATACGCGTTACGCCGATAGCGTTA
AAGGTCGCTTTACCATTTCTGCAGACACCAGTAAAAACACGGCTTATCTGCAGATGAATTCTCT
GCGTGCGGAAGACACGGCCGTGTATTACTGCAGTCGCTGGGGCGGTGATGGCTTTTATGCAATG
GACTACTGGGGCCAGGGTACCCTGGTTACGGTCAGCTCTGGATCCCATCACCATCACCATCACT
AA

Herceptin Fab heavy chain
ATGGAAGTCCAACTGGTCGAATCGGGTGGTGGTCTGGTTCAGCCGGGCGGTTCACTGCGTCTGT
CGTGTGCTGCCTCGGGTTTCAACATCAAAGATACCTATATTCATTGGGTGCGTCAGGCCCCGGG
TAAAGGTCTGGAATGGGTTGCCCGCATCTACCCGACCAACGGCTATACGCGTTACGCCGATAGC
GTTAAAGGTCGCTTTACCATTTCTGCAGACACCAGTAAAAACACGGCTTATCTGCAGATGAATT

CTCTGCGTGCGGAAGACACGGCCGTGTATTACTGCAGTCGCTGGGGCGGTGATGGCTTTTATGC
AATGGACTACTGGGGCCAGGGTACCCTGGTTACGGTCAGCTCTGCTAGCACCAAAGGCCCGTC
AGTTTTCCCGCTGGCCCCGAGTTCCAAATCGACCAGCGGCGGTACCGCCGCACTGGGTTGTCTG
GTGAAAGATTATTTTCCGGAACCGGTGACCGTTTCCTGGAACTCAGGCGCACTGACCAGCGGCG
TGCACACCTTCCCGGCTGTCCTGCAGTCATCGGGCCTGTATTCTCTGAGCTCTGTGGTTACCGTC
CCGAGTTCCTCACTGGGTACCCAAACGTACATTTGCAACGTGAATCATAAACCGTCCAATACGA
AAGTGGATAAGAAAGTGGAACCGCCGAAATCATGTGACAAAACCGGATCCCATCACCATCACC
ATCACTAA

Herceptin Fab light chain
ATGGATATTCAGATGACCCAGAGCCCGTCCAGCCTGTCAGCAAGCGTTGGCGACCGTGTTACGA
TTACCTGCCGTGCCTCGCAAGATGTGAACACCGCGGTGGCCTGGTATCAGCAAAAACCGGGCA
AAGCACCGAAACTGCTGATTTATAGTGCTTCCTTTCTGTACTCTGGTGTTCCGTCCCGTTTCTCA
GGCTCGCGCAGCGGTACCGATTTTACCCTGACGATCAGCTCTCTGCAGCCGGAAGACTTCGCCA
CGTATTACTGCCAGCAACACTACACGACCCCGCCGACCTTCGGCCAGGGCACCAAAGTGGAAA
TCAAACGCACCGTCGCGGCGCCGTCGGTCTTTATCTTCCCGCCGAGCGATGAACAACTGAAATC
TGGTACCGCGAGTGTGGTTTGTCTGCTGAACAATTTTTATCCGCGTGAAGCGAAAGTCCAGTGG
AAGGTGGACAACGCCCTGCAGTCTGGCAATAGTCAAGAATCCGTGACCGAACAAGATTCAAAA
GACTCGACGTACAGCCTGAGTTCCACCCTGACGCTGAGCAAGGCAGATTATGAAAAACATAAG
GTGTACGCTTGCGAAGTTACCCACCAGGGTCTGTCCCTGCCGGTTACGAAATCATTCAACCGCG
GCGAATGTTAA

Tysabri scFv
ATGGACATCCAGATGACCCAGAGCCCGTCCAGCCTGAGCGCAAGCGTCGGCGACCGTGTTACG
ATTACGTGCAAAACCAGCCAAGACATTAACAAATATATGGCGTGGTACCAGCAAACCCCGGGT
AAAGCCCCGGAACTGCTGATTCATTATACGTCGGCACTGCAGCCGGGCATCCCGTCTCGTTTTA
GTGGCTCCGGTTCAGGCCGCGATTACACCTTCACGATTAGCTCTCTGCAACCGGAAGACATCGC
GACCTATTACTGCCTGCAGTATGACAATCTGTGGACCTTCGGCCAGGGCACCAAAGTGGAAATC
AAAGGCGCCAGCGGCGGTGGCGGTTCTGGTGGCGGTGGCAGCGGTGGCGGTGGCTCGAGCCAG
GTCCAACTGGTCCAATCAGGTGCCGAAGTCAAAAAACCGGGCGCATCCGTCAAAGTGTCGTGC
AAAGCGAGTGGCTTCAACATCAAAGATACGTATATTCATTGGGTTCGTCAGGCACCGGGTCAAC
GCCTGGAATGGATGGGCCGTATCGATCCGGCTAACGGTTATACCAAATACGACCCGAAATTTCA
GGGCCGCGTGACCATTACGGCGGATACGTCTGCAAGTACCGCTTATATGGAACTGAGCTCTCTG
CGCAGCGAAGACACCGCGGTGTATTACTGCGCCCGTGAAGGCTATTACGGTAATTATGGCGTTT
ACGCCATGGATTACTGGGGCCAGGGTACGCTGGTTACCGTCAGTTCCGGATCCCATCACCATCA
CCATCACTAA

Tysabri Fab heavy chain
ATGCAGGTCCAACTGGTCCAATCAGGTGCCGAAGTCAAAAAACCGGGCGCATCCGTCAAAGTG
TCGTGCAAAGCGAGTGGCTTCAACATCAAAGATACGTATATTCATTGGGTTCGTCAGGCACCGG
GTCAACGCCTGGAATGGATGGGCCGTATCGATCCGGCTAACGGTTATACCAAATACGACCCGA
AATTTCAGGGCCGCGTGACCATTACGGCGGATACGTCTGCAAGTACCGCTTATATGGAACTGAG
CTCTCTGCGCAGCGAAGACACCGCGGTGTATTACTGCGCCCGTGAAGGCTATTACGGTAATTAT
GGCGTTTACGCCATGGATTACTGGGGCCAGGGTACGCTGGTTACCGTCAGTTCCGCTAGCACCA
AAGGTCCGAGTGTTTTTCCGCTGGCCCCGTGCTCCCGTTCAACGTCGGAAAGCACCGCGGCCCT
GGGTTGTCTGGTGAAAGATTATTTTCCGGAACCGGTGACCGTTTCCTGGAACTCAGGTGCACTG
ACGTCTGGCGTTCATACCTTCCCGGCTGTCCTGCAGTCATCGGGCCTGTATAGTCTGAGCTCTGT
GGTTACGGTCCCGAGTTCCTCACTGGGTACCAAAACGTACACCTGCAACGTGGATCACAAACCG
AGCAATACCAAAGTGGACAAACGCGTTGAATCTAAATATGGTGGATCCCATCACCATCACCAT
CACTAA

Tysabri Fab light chain
ATGGACATCCAGATGACCCAGAGCCCGTCCAGCCTGAGCGCAAGCGTCGGCGACCGTGTTACG
ATTACGTGCAAAACCAGCCAAGACATTAACAAATATATGGCGTGGTACCAGCAAACCCCGGGT
AAAGCCCCGGAACTGCTGATTCATTATACGTCGGCACTGCAGCCGGGCATCCCGTCTCGTTTTA

GTGGCTCCGGTTCAGGCCGCGATTACACCTTCACGATTAGCTCTCTGCAACCGGAAGACATCGC
GACCTATTACTGCCTGCAGTATGACAATCTGTGGACCTTCGGCCAGGGCACCAAAGTGGAAATC
AAACGCACCGTGGCGGCGCCGTCGGTCTTTATCTTCCCGCCGAGCGATGAACAACTGAAATCTG
GTACCGCGAGTGTGGTTTGTCTGCTGAACAATTTTTATCCGCGTGAAGCGAAAGTCCAGTGGAA
GGTGGACAACGCCCTGCAGTCTGGCAATAGTCAAGAATCCGTGACCGAACAAGATTCAAAAGA
CTCGACGTACAGCCTGAGTTCCACCCTGACGCTGAGCAAGGCAGATTATGAAAAACATAAGGT
GTACGCTTGCGAAGTTACCCACCAGGGTCTGTCCCTGCCGGTTACGAAATCATTCAACCGCGGC
GAATGTTAA

Mab123 scFv
ATGGATATTGTTCTGACGCAGTCTCCGTCCTCTCTGTCGGCTTCCCTGGGCGATACGATTACGAT
TACCTGCCATGCGTCACAGAACATTAATGTTTGGCTGTCGTGGTATCAGCAAAAACCGGGTAAC
ATTCCGAAACTGCTGATCTACAAGGCCAGCAATCTGCACACGGGCGTCCCGTCTCGTTTTAGCG
GTTCTGGCAGTGGTACCGGCTTCACCCTGACGATTAGCTCTCTGCAGCCGGAAGATATCGCAAC
CTATTACTGCCAGCAAGGTCAAAGCTATCCGCTGACGTTTGGCGGTGGCACCAAACTGGAAATT
AAGGGCGCCAGCGGCGGTGGCGGTTCTGGTGGCGGTGGCAGCGGTGGCGGTGGCTCGAGCGAA
GTCAAACTGCAAGAATCAGGTGGCGGTCTGGTTCAGCCGGGTGGTAGTCTGAAACTGTCCTGCG
CAACCTCAGGCTTTACGTTCTCCGATTATTACATGTATTGGGTTCGTCAGACCCCGGAAAAACG
CCTGGAATGGGTGGCTTACATTTCTAACGGCGGTGGCAGTACCTATTACCCGGATACGGTGAAA
GGTCGTTTTACCATCAGCCGCGACAACGCGAAGAATACGCTGTATCTGCAGATGTCGCGTCTGA
AAAGCGAAGATACCGCGATGTATTACTGTGCCCGTCATGGCGGCTATTACGCGATGGATTATTG
GGGTCAAGGCACCACGGTTACCGTCAGCTCTGGATCCCATCACCATCACCATCACTAA

Mab123 Fab heavy chain
ATGGAAGTCAAACTGCAAGAATCAGGTGGCGGTCTGGTTCAGCCGGGTGGTAGTCTGAAACTG
TCCTGCGCAACCTCAGGCTTTACGTTCTCCGATTATTACATGTATTGGGTTCGTCAGACCCCGGA
AAAACGCCTGGAATGGGTGGCTTACATTTCTAACGGCGGTGGCAGTACCTATTACCCGGATACG
GTGAAAGGTCGTTTTACCATCAGCCGCGACAACGCGAAGAATACGCTGTATCTGCAGATGTCGC
GTCTGAAAAGCGAAGATACCGCGATGTATTACTGTGCCCGTCATGGCGGCTATTACGCGATGGA
TTATTGGGGTCAAGGCACCACGGTTACCGTCAGCTCTGCAAAAACCACGGCTCCGTCTGTGTAC
CCGCTGGCACCGGTTTGCGGTGATACCACGGGCAGTTCCGTCACCCTGGGTTGTCTGGTGAAAG
GCTATTTTCCGGAACCGGTGACCCTGACGTGGAACAGCGGTAGTCTGTCATCGGGCGTTCATAC
CTTCCCGGCGGTCCTGCAGAGTGATCTGTACACGCTGAGCTCTAGTGTGACCGTTACGTCCTCA
ACCTGGCCGTCCCAATCAATTACGTGCAACGTCGCACACCCGGCGAGCAGCACCAAAGTGGAC
AAAAAGATTGAACCGCGTGGCCATCACCATCACCATCACTAA

Mab123 Fab light chain
ATGGATATTGTTCTGACGCAGTCTCCGTCCTCTCTGTCGGCTTCCCTGGGCGATACGATTACGAT
TACCTGCCATGCGTCACAGAACATTAATGTTTGGCTGTCGTGGTATCAGCAAAAACCGGGTAAC
ATTCCGAAACTGCTGATCTACAAGGCCAGCAATCTGCACACGGGCGTCCCGTCTCGTTTTAGCG
GTTCTGGCAGTGGTACCGGCTTCACCCTGACGATTAGCTCTCTGCAGCCGGAAGATATCGCAAC
CTATTACTGCCAGCAAGGTCAAAGCTATCCGCTGACGTTTGGCGGTGGCACCAAACTGGAAATT
AAGCGCGCAGATGCAGCACCGACGGTGTCTATCTTCCCGCCGAGTTCCGAACAGCTGACCAGT
GGTGGCGCTTCCGTGGTTTGTTTTCTGAACAACTTCTACCCGAAGGATATCAACGTCAAGTGGA
AGATCGACGGTAGTGAACGTCAGAACGGCGTGCTGAACAGCTGGACGGATCAAGACTCGAAAG
ATAGCACCTACTCTATGTCATCGACCCTGACGCTGACCAAGGACGAATATGAACGTCATAATAG
TTACACGTGCGAAGCCACCCACAAAACGTCCACCTCACCGATCGTTAAGAGCTTTAACCGCAAT
GAATGTTAA

3M8O scFv
ATGGATATCGTTATGACCCAGAGTCCGCTGAGCCTGTCCGTCACCCCGGGCGAACCGGCGAGC
ATTTCCTGTCGTTCTTCACAAAGCCTGCTGCGTCGCGATGGTCATAACGACCTGGAATGGTATCT
GCAGAAACCGGGCCAGAGCCCGCAACCGCTGATTTATCTGGGTTCCACCCGTGCGAGCGGCGT
GCCGGATCGCTTTAGCGGCTCTGGTAGTGGCACCGACTTCACGCTGAAAATTATCCGTGTTGAA
GCGGAAGATGCCGGCACGTATTACTGCATGCAAAATAAACAAACCCCGCTGACCTTCGGCCAG

GGCACCCGTCTGGAAATCAAAGGCGCCAGCGGCGGTGGCGGTTCTGGTGGCGGTGGCAGCGGT
GGCGGTGGCTCGAGCGAAGTCCAACTGGTCGAATCGGGCGGTGGTCTGGTGCAGCCGGGTGGC
AGCCTGAAACTGTCATGTGCGGCAAGCGGTTTTACCCTGAGTGGCTCCAACGTCCATTGGGTGC
GTCAGGCGTCTGGCAAAGGTCTGGAATGGGTGGGTCGTATTAAACGCAATGCCGAAAGCGATG
CAACCGCTTATGCGGCCTCTATGCGTGGCCGCCTGACGATCAGCCGCGATGACTCTAAAAACAC
CGCGTTTCTGCAGATGAATAGCCTGAAATCTGATGACACCGCCATGTATTACTGCGTCATTCGT
GGTGATGTGTACAACCGCCAGTGGGGCCAAGGTACCCTGGTCACCGTGAGCTCTGGATCCCATC
ACCATCACCATCACTAA

3M8O Fab heavy chain
ATGGAAGTCCAACTGGTCGAATCGGGCGGTGGTCTGGTGCAGCCGGGTGGCAGCCTGAAACTG
TCATGTGCGGCAAGCGGTTTTACCCTGAGTGGCTCCAACGTCCATTGGGTGCGTCAGGCGTCTG
GCAAAGGTCTGGAATGGGTGGGTCGTATTAAACGCAATGCCGAAAGCGATGCAACCGCTTATG
CGGCCTCTATGCGTGGCCGCCTGACGATCAGCCGCGATGACTCTAAAAACACCGCGTTTCTGCA
GATGAATAGCCTGAAATCTGATGACACCGCCATGTATTACTGCGTCATTCGTGGTGATGTGTAC
AACCGCCAGTGGGGCCAAGGTACCCTGGTCACCGTGAGCTCTGCTAGCCCGACGTCCCCGAAA
GTGTTTCCGCTGTCACTGTGCTCGACCCAGCCGGACGGCAACGTGGTTATCGCCTGTCTGGTTC
AGGGCTTTTTCCCGCAAGAACCGCTGAGCGTGACGTGGTCAGAATCGGGCCAGGGTGTTACCG
CACGTAATTTCCCGCCGAGTCAAGATGCTTCCGGCGACCTGTATACCACGAGTTCCCAGCTGAC
CCTGCCGGCCACCCAATGCCTGGCAGGTAAAAGCGTGACCTGTCATGTTAAACACTACACGAA
CCCGAGTCAGGATGTTACCGTCCCGTGCCCGGTTCCGTCCACCGGATCCCATCACCATCACCAT
CACTAA

3M8O Fab light chain
ATGGATATCGTTATGACCCAGAGTCCGCTGAGCCTGTCCGTCACCCCGGGCGAACCGGCGAGC
ATTTCCTGTCGTTCTTCACAAAGCCTGCTGCGTCGCGATGGTCATAACGACCTGGAATGGTATCT
GCAGAAACCGGGCCAGAGCCCGCAACCGCTGATTTATCTGGGTTCCACCCGTGCGAGCGGCGT
GCCGGATCGCTTTAGCGGCTCTGGTAGTGGCACCGACTTCACGCTGAAAATTATCCGTGTTGAA
GCGGAAGATGCCGGCACGTATTACTGCATGCAAAATAAACAAACCCCGCTGACCTTCGGCCAG
GGCACCCGTCTGGAAATCAAACGCACCGTGGCGGCGCCGTCGGTCTTTATCTTCCCGCCGAGCG
ATGAACAACTGAAATCTGGTACCGCGAGTGTGGTTTGTCTGCTGAACAATTTTTATCCGCGTGA
AGCGAAAGTCCAGTGGAAGGTGGACAACGCCCTGCAGTCTGGCAATAGTCAAGAATCCGTGAC
CGAACAAGATTCAAAAGACTCGACGTACAGCCTGAGTTCCACCCTGACGCTGAGCAAGGCAGA
TTATGAAAAACATAAGGTGTACGCTTGCGAAGTTACCCACCAGGGTCTGTCCCTGCCGGTTACG
AAATCATTCAACCGCGGCGAATGTTAA

2R56 scFv
ATGGACATTGTTATGACCCAGTCCCCGTCGTCCCTGAGCGCAAGCGTCGGTGATCGCGTGACCA
TTACCTGCCGTGCATCGCAAGGCATCAGCTCTCGTCTGGCATGGTATCAGCAAAAACCGGGTAA
AGCTCCGAAACTGCTGATTTATGCCGCCAGTTCCCTGCAGAGCGGCGTGCCGAGTCGCTTTAGC
GGCTCTGGTTCCGGCACCGAATTCACCCTGACGATCTCATCGCTGCAACCGGAAGATTTTGCCA
CGTATTACTGCCAGCAATACCATTCGTATCCGTGGACCTTCGGTCAGGGCACCAAACTGGAAAT
CAAAGGCGCCAGCGGCGGTGGCGGTTCTGGTGGCGGTGGCAGCGGTGGCGGTGGCTCGAGCCA
GGTGTCACTGCGCGAATCGGGTGGTGGTCTGGTCCAACCGGGTCGTTCACTGCGTCTGTCATGT
ACGGCGTCGGGCTTTACCTTTCGTCATCATGGTATGACCTGGGTTCGTCAGGCCCCGGGTAAAG
GCCTGGAATGGGTCGCCAGCCTGTCTGGTAGTGGCACCAAAACGCATTTTGCAGATTCCGTGAA
AGGCCGTTTCACGATTTCCCGCGACAACTCAAACAATACCCTGTATCTGCAGATGGATAATGTT
CGTGATGAAGACACCGCAATCTATTACTGCGCAAAAGCTAAACGTGTCGGTGCTACCGGCTACT
TTGATCTGTGGGGTCGTGGTACCCTGGTCACCGTGAGCTCTGGATCCCATCACCATCACCATCA
CTAA

2R56 Fab heavy chain
ATGCAGGTGTCACTGCGCGAATCGGGTGGTGGTCTGGTCCAACCGGGTCGTTCACTGCGTCTGT
CATGTACGGCGTCGGGCTTTACCTTTCGTCATCATGGTATGACCTGGGTTCGTCAGGCCCCGGG
TAAAGGCCTGGAATGGGTCGCCAGCCTGTCTGGTAGTGGCACCAAAACGCATTTTGCAGATTCC

GTGAAAGGCCGTTTCACGATTTCCCGCGACAACTCAAACAATACCCTGTATCTGCAGATGGATA
ATGTTCGTGATGAAGACACCGCAATCTATTACTGCGCAAAAGCTAAACGTGTCGGTGCTACCGG
CTACTTTGATCTGTGGGGTCGTGGTACCCTGGTCACCGTGAGCTCTGCTAGCACGCAGTCCCCG
TCAGTGTTCCCGCTGACCCGCTGCTGTAAAAACATTCCGTCGAATGCAACCAGCGTTACGCTGG
GTTGTCTGGCTACCGGCTATTTTCCGGAACCGGTTATGGTCACCTGGGATACGGGTTCTCTGAAT
GGTACCACCATGACCCTGCCGGCCACCACCCTGACCCTGAGTGGTCATTACGCCACCATCTCTC
TGCTGACCGTCAGTGGTGCGTGGGCCAAACAGATGTTTACCTGTCGCGTGGCCCACACCCCGAG
TTCCACGGATTGGGTGGACAACAAAACCTTTTCGGTTTGTAGCCGCGACTTCACCGGATCCCAT
CACCATCACCATCACTAA

2R56 Fab light chain
ATGGACATTGTTATGACCCAGTCCCCGTCGTCCCTGAGCGCAAGCGTCGGTGATCGCGTGACCA
TTACCTGCCGTGCATCGCAAGGCATCAGCTCTCGTCTGGCATGGTATCAGCAAAAACCGGGTAA
AGCTCCGAAACTGCTGATTTATGCCGCCAGTTCCCTGCAGAGCGGCGTGCCGAGTCGCTTTAGC
GGCTCTGGTTCCGGCACCGAATTCACCCTGACGATCTCATCGCTGCAACCGGAAGATTTTGCCA
CGTATTACTGCCAGCAATACCATTCGTATCCGTGGACCTTCGGTCAGGGCACCAAACTGGAAAT
CAAACGCACCGTGGCGGCGCCGTCGGTCTTTATCTTCCCGCCGAGCGATGAACAACTGAAATCT
GGTACCGCGAGTGTGGTTTGTCTGCTGAACAATTTTTATCCGCGTGAAGCGAAAGTCCAGTGGA
AGGTGGACAACGCCCTGCAGTCTGGCAATAGTCAAGAATCCGTGACCGAACAAGATTCAAAAG
ACTCGACGTACAGCCTGAGTTCCACCCTGACGCTGAGCAAGGCAGATTATGAAAAACATAAGG
TGTACGCTTGCGAAGTTACCCACCAGGGTCTGTCCCTGCCGGTTACGAAATCATTCAACCGCGG
CGAATGTTAA

1QLR scFv
ATGGAAATCGTGCTGACGCAATCGCCGGCAACGCTGTCCCTGTCTCCGGGTGAACGCGCAACG
CTGTCGTGTGGTGCAAGCCAATCGGTTAGCTCTAACTATCTGGCATGGTACCAGCAAAAACCGG
GTCAGGCTCCGCGTCTGCTGATTTATGATGCGAGTTCCCGTGCCACCGGCATCCCGGACCGCTT
TTCAGGCTCGGGTAGCGGCACGGATTTCACCCTGACGATTAGTCGCCTGGAACCGGAAGACTTT
GCCGTGTATTACTGCCAGCAATATGGTAGCAGTCCGCTGACCTTCGGTGGTGGCACGAAAGTGG
AAATCAAAGGCGCCAGCGGCGGTGGCGGTTCTGGTGGCGGTGGCAGCGGTGGCGGTGGCTCGA
GCGAAGTCCAACTGCAACAATGGGGCGCTGGCCTGCTGAAACCGTCGGAAACCCTGTCCCTGA
CCTGTGCCGTGTATGGTGGCTCGTTCAGCGATTATTACTGGTCTTGGATTCGTCAGCCGCCGGGC
AAAGGTCTGGAATGGATTGGCGAAATCAACCATTCAGGTTCGACCAACTATAATCCGAGTCTG
AAATCCCGCGTTACCATCAGCGTCGATACGTCTAAAAATCAATTTAGCCTGAAACTGAGCTCTG
TTACCGCGGCCGATACGGCTGTCTATTACTGTGCCCGTCCGCCGCATGACACCAGCGGTCACTA
TTGGAACTACTGGGGCCAGGGTACGCTGGTTACCGTCAGTTCCGGATCCCATCACCATCACCAT
CACTAA

1QLR Fab heavy chain
ATGGAAGTCCAACTGCAACAATGGGGCGCTGGCCTGCTGAAACCGTCGGAAACCCTGTCCCTG
ACCTGTGCCGTGTATGGTGGCTCGTTCAGCGATTATTACTGGTCTTGGATTCGTCAGCCGCCGG
GCAAAGGTCTGGAATGGATTGGCGAAATCAACCATTCAGGTTCGACCAACTATAATCCGAGTCT
GAAATCCCGCGTTACCATCAGCGTCGATACGTCTAAAAATCAATTTAGCCTGAAACTGAGCTCT
GTTACCGCGGCCGATACGGCTGTCTATTACTGTGCCCGTCCGCCGCATGACACCAGCGGTCACT
ATTGGAACTACTGGGGCCAGGGTACGCTGGTTACCGTCAGTTCCGGTAGCGCTAGCGCCCCGAC
CCTGTTTCCGCTGGTGTCTTGCGAAAATAGTCCGTCCGATACGTCATCGGTGGCCGTTGGCTGTC
TGGCACAGGATTTTCTGCCGGACAGTATCACCTTCTCCTGGAAATACAAAAACAACTCAGACAT
CAGCTCTACGCGTGGTTTCCCGTCGGTTCTGCGCGGCGGTAAATACGCAGCTACCTCACAAGTG
CTGCTGCCGTCGAAAGATGTTATGCAGGGCACCGACGAACATGTGGTTTGCAAAGTGCAGCAC
CCGAACGGTAATAAAGAGAAAAACGTGCCGCTGCCGGTTGGATCCCATCACCATCACCATCAC
TAA

1QLR Fab light chain
ATGGAAATCGTGCTGACGCAATCGCCGGCAACGCTGTCCCTGTCTCCGGGTGAACGCGCAACG
CTGTCGTGTGGTGCAAGCCAATCGGTTAGCTCTAACTATCTGGCATGGTACCAGCAAAAACCGG

GTCAGGCTCCGCGTCTGCTGATTTATGATGCGAGTTCCCGTGCCACCGGCATCCCGGACCGCTT
TTCAGGCTCGGGTAGCGGCACGGATTTCACCCTGACGATTAGTCGCCTGGAACCGGAAGACTTT
GCCGTGTATTACTGCCAGCAATATGGTAGCAGTCCGCTGACCTTCGGTGGTGGCACGAAAGTGG
AAATCAAACGCACGGTGGCGGCGCCGTCGGTCTTTATCTTCCCGCCGAGCGATGAACAACTGA
AATCTGGTACCGCGAGTGTGGTTTGTCTGCTGAACAATTTTTATCCGCGTGAAGCGAAAGTCCA
GTGGAAGGTGGACAACGCCCTGCAGTCTGGCAATAGTCAAGAATCCGTGACCGAACAAGATTC
AAAAGACTCGACGTACAGCCTGAGTTCCACCCTGACGCTGAGCAAGGCAGATTATGAAAAACA
TAAGGTGTACGCTTGCGAAGTTACCCACCAGGGTCTGTCCCTGCCGGTTACGAAATCATTCAAC
CGCGGCGAATGTTAA
